# Current State of Vascular Resections in Pancreatic Cancer Surgery

**DOI:** 10.1155/2015/120207

**Published:** 2015-11-02

**Authors:** Thilo Hackert, Lutz Schneider, Markus W. Büchler

**Affiliations:** Department of Surgery, University of Heidelberg, Im Neuenheimer Feld 110, 69120 Heidelberg, Germany

## Abstract

Pancreatic cancer (PDAC) is the fourth leading cause of cancer-related mortality in the Western world and, even in 2014, a therapeutic challenge. The only chance for long-term survival is radical surgical resection followed by adjuvant chemotherapy which can be performed in about 20% of all PDAC patients by the time of diagnosis. As pancreatic surgery has significantly changed during the past years, extended operations, including vascular resections, have become more frequently performed in specialized centres and the border of resectability has been pushed forward to achieve a potentially curative approach in the respective patients in combination with neoadjuvant and adjuvant treatment strategies. In contrast to adjuvant treatment which has to be regarded as a cornerstone to achieve long-term survival after resection, neoadjuvant treatment strategies for locally advanced findings are currently under debate. This overview summarizes the possibilities and evidence of vascular, namely, venous and arterial, resections in PDAC surgery.

## 1. Introduction

Overall long-term survival rates of 1–5% are associated with pancreatic cancer (PDAC) underlining the poor prognosis of this tumor entity [1, 2]. The major obstacle to improve this situation is the fact that PDAC diagnosis is made late and in an advanced tumor stage in the majority of patients precluding them from surgical resection due to distant spread of the disease. Approximately 10–20% of PDAC patients can consequently undergo potentially curative surgery aiming at a radical R0 resection which results in long-term 5-year survival rates of 20–25% [3, 4]. As pancreatic surgery is challenging with regard to preoperative diagnostic, surgical procedures as well as postoperative care and complication management, the value of centralization of pancreatic surgery in centres of excellence and high volume institutions is unquestionable today and has been demonstrated in numerous studies the last 15 years [5, 6]. In this setting, implying experience of the individual surgeon who continuously performs pancreatic resections and the environment with an interdisciplinary team of specialists to optimize perioperative care including ICU treatment and complication management, mortality rates following major pancreatic resections below 5% are standard today [5, 6]. In this context, the borders of resectability have been pushed and extended surgical approaches in PDAC have become commonly performed, which include vascular as well as multivisceral resections [7, 8]. This has been accompanied by scientific work-up of the results of these operations in terms of surgical as well as oncological outcome resulting in an increasing number of publications and a meanwhile satisfying level of evidence with a relevant influence on recent national and international guidelines consensus statements [9, 10]. The present review gives an overview on the development and current state of venous and arterial resections in PDAC surgery.

## 2. Evaluation of Resectability and Borderline Resectability

**Figure 1:**
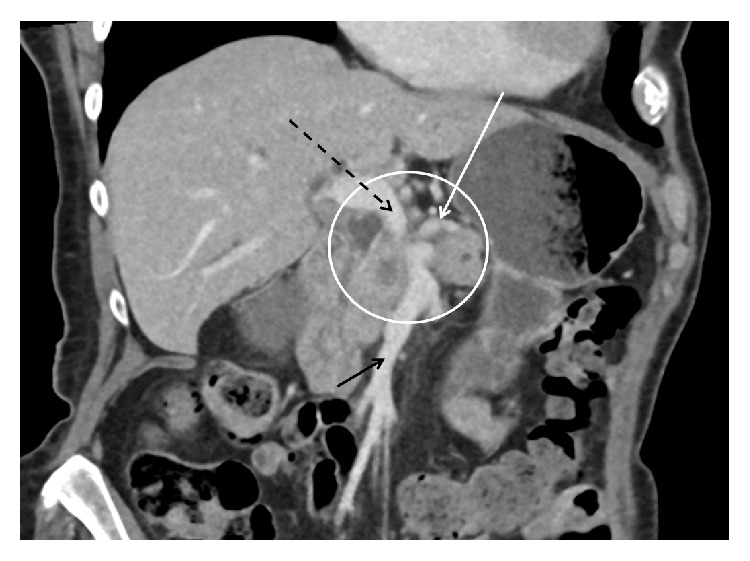
CT scan (coronary reconstruction) showing PDAC tumor infiltration of the portal vein confluence (white circle). Superior mesenteric vein (black arrow), portal vein (broken black arrow), and splenic vein (white arrow) without thrombosis, adequate diameter of the portal, and superior mesenteric vein to perform an end-to- end anastomosis.

Situations in which vascular resections are required are often described as “borderline resectable” findings. In 2014, the International Study Group for Pancreatic Surgery (ISGPS) has published a consensus statement to standardize the definition of borderline resectability in accordance with the guidelines of the National Comprehensive Cancer Network (NCCN) as well as the definition of extended resections [11, 12]. Following these recommendations, preoperative evaluation of resectability should be based on a computed tomography (CT) scan with a pancreas-specific protocol, for example, a “hydropancreas” CT, as this offers best local resolution with regard to tumor extension and infiltration towards the vascular structures (Figure 1). Three grades of resectability can be defined for localized PDAC which are termed as “resectable,” “borderline resectable,” and “irresectable” [11]. While a resectable tumor has no vascular attachment (no distortion of the venous structures and clearly preserved fat planes towards the arteries), borderline resectability is defined as distortion/narrowing/occlusion of the mesentericoportal veins with a technical possibility of reconstruction on the proximal and distal margin of the veins. Furthermore, a semicircumferential abutment (≤180°) of the superior mesenteric artery (SMA) or an attachment at the hepatic artery without the celiac axis is regarded as a borderline resectable finding. Consequently, irresectability is defined as a more extended involvement of the SMA, the celiac axis, aorta or inferior vena cava. Furthermore, involvement of the mesentericoportal venous system can fulfill the criteria of irresectability if there is no technical possibility for reconstruction, for example, in case of tumor-associated portal cavernous transformation.

Regarding the performance of resections, borderline findings in venous and arterial vessel involvement have to be differentiated. In venous borderline resectability, no neoadjuvant treatment is recommended, instead upfront surgery should be performed and, if the intraoperative finding matches the presumed borderline situation as defined above, completed as an en bloc tumor removal with venous replacement [11, 12]. In contrast, when suspected arterial borderline resectability is intraoperatively confirmed as a true arterial involvement, no general recommendation for resection is given, but palliative treatment should be regarded as the standard of care. In individual decision, however, these recommendations may be modified and neoadjuvant treatment with a consecutive reexploration and eventually resection is possible as well as direct arterial resection in exceptional cases or under study conditions.

Restaging after neoadjuvant treatment may be challenging, as the differentiation of vital tumor and fibrosis by conventional cross-sectional imaging is limited and even PET-CT scans do not offer 100% accuracy [27]. Therefore, in cases of clear tumor regression, a surgical exploration should be performed as well as in patients showing stable disease status after completion of the neoadjuvant treatment. The rationale for this is the fact that, despite still visible soft tissue, this is often found to be only fibrotic residual changes [28]. In these cases, after confirming absence of vital tumor, a sharp dissection without vascular resection is possible and eventually a ypT0 situation may be found. Patients with a clear tumor progression under neoadjuvant treatment should be excluded from secondary exploration. Due to the three scenarios described, the neoadjuvant treatment is helpful to stratify patients and recognize those with borderline findings who do not benefit from extended resections.

## 3. Venous Resections

En bloc vascular resections during pancreatoduodenectomy (PD) to achieve tumor clearance and improve survival in case of portomesenteric vein involvement were published more than 30 years ago [29]. From these anecdotal reports, the technical feasibility was concluded; however, it took another two decades until these approaches gained widespread acceptance in centres around the world. Besides venous resection in PD, this approach is also performed during distal (DP) or total pancreatectomy (TP) [30–32]. However, especially in TP, several aspects regarding venous drainage of the stomach have to be respected when the portal, and usually also the splenic vein, is resected, which is mentioned in detail below.

The site of tumor infiltration of the vein has to be carefully evaluated already in the preoperative cross-sectional imaging. Proximal tumor adherence or infiltration can usually be handled more easily, as the vessel diameter is large enough to create a sufficient anastomosis. In case of a more distally located tumor infiltration far below the mesenterico-splenic confluence, the decreasing vascular diameter of the superior mesenteric vein may limit the technical possibility to perform a venous resection [11].

Regarding surgical technique, venous resections can be performed differently depending on the location and length of tumor adherence. A latero-tangential resection of the portal vein is possible when tumor infiltration reaches the vein from the right circumference and can be excised with a small patch and direct closure of the defect directly without a hemodynamically relevant stenosis [30–32]. Adequate venous drainage of the small bowel needs to be ensured afterwards by direct flow measurement and the clinical evaluation of the perfusion aspect of the intestine during the remaining operation time, which is usually long enough to recognize venous congestion if present.

When a tangential vein resection is not possible, the mesenteric root should be mobilized completely by resolving the attachment of the right hemicolon to the retroperitoneal adhesions [33]. This gives a great flexibility of the mesenteric vein and almost always allows approximation of the distal and proximal resection margins of the vein without any critical tension. Following resection of the tumor-bearing venous segment, continuity of the vessel can be restored by a direct end-to-end anastomosis as the corresponding diameters of the distal and proximal lumen can usually be adapted without causing any obstruction to the free venous bowel drainage.

In case the resected venous length cannot be bridged by the direct anastomosis, a vascular graft needs to be inserted. For this purpose, autologous grafting (e.g., renal vein and saphenous vein) is possible but requires a venous harvesting before clamping and resection [34]. Alternatively, synthetic grafts, for example, a ringed goretex prosthesis, can be chosen to bridge the resected vein segment. The insertion of a synthetic graft always implies the possible problems artificial material may cause in case of infection or anastomotic leakage. A situation of a synthetic graft in combination with a pancreatic fistula must be regarded as a high risk constellation for postpancreatectomy hemorrhage or difficult to treat long-lasting graft infection. Yet, the clinical impact of this complication seems to be rather small; in a series of 110 patients undergoing venous resection with different reconstruction techniques no difference in surgical outcome was shown when different types of venous reconstruction (venorrhaphy, end-to-end anastomosis, and graft insertion) were observed [33].

When other surgical outcome parameters are considered, it has been demonstrated that both, resection with a direct anastomosis or the interposition of a graft, can be performed safely. Large series showed that surgical morbidity and mortality rates are comparable to standard pancreatic head resections [33–35]. This has been scientifically examined and confirmed in two recently published systematic reviews [36, 37]. In the review by Siriwardana and Siriwardena [36], 52 manuscripts with 6333 patients in whom pancreatic resection was performed for PDAC were included. 1646 of these patients (26%) underwent synchronous porto-superior mesenteric vein resection mainly together with partial pancreatoduodenectomy (71%) or total pancreatectomy (24%). Median operation time was 8.5 hours, median blood loss 1750 mL, overall morbidity 42% (9 to 78%), and perioperative mortality 5.9%. The more recent meta-analysis by Zhou et al. [37] included 19 studies and 661 patients with venous resections during PDAC resections that were compared to 2247 patients undergoing similar operation without vessel resection. Both groups were characterized by comparable surgical outcome. Furthermore, in terms of oncological results, no difference in overall survival between both patient collectives was found, resulting in a 5-year survival rate of 12.3%, certainly superior to palliative treatment.  Table 1 gives an overview of the largest studies on venous resections in PDAC.

A special aspect in venous resections that has to be respected in certain situations is the patency of venous gastric drainage. In PD, the splenic vein can be closed during venous resection as the stomach is usually drained sufficiently via the coronary vein (if preserved) and collaterals via the short gastric veins.

In contrast, in TP for PDAC, which is usually combined with splenectomy, venous resections may cause severe disturbances of the gastric drainage, especially when the coronary vein is removed during resection. In this situation, two scenarios may occur. On one hand a venous congestion of the remaining stomach may require a classical distal or even subtotal stomach resection to avoid ischemic complications with either long-lasting delayed gastric emptying or even necrosis of the stomach with the need for a reintervention and consecutive stomach resection. On the other hand, if the resected coronary vein can technically be preserved, there is the possibility for reinsertion into the mesentericoportal axis to restore stomach drainage and avoid any type of gastrectomy [38].

In addition to PD and venous resection alone, also multivisceral approaches established procedures to achieve a radical tumor removal [24, 31, 32]. Although these are associated with an increased morbidity, perioperative mortality and long-term survival are not negatively influenced in these patients [31, 32]. In approximately 20% of the patients, multivisceral resection is performed together with portal or superior mesenteric vein resections. This additional procedure does not increase the risk for complications and should therefore be performed in patients qualifying for an extended approach of complete tumor removal [24].

In conclusion, venous resections during surgery for pancreatic cancer can therefore be regarded as a standard procedure in experienced hands and should be performed in a routine setting to achieve a complete removal of the tumor, which has meanwhile been generally accepted and is explicitly stated in national and international guidelines such as the German and the ISGPS consensus statements [9, 10].

## 4. Arterial Resections

The resection of the celiac axis or the superior mesenteric artery has occasionally been performed since the 1970s in selected patients but is still regarded as an extraordinary approach in PDAC surgery [31, 32, 39–42]. Arterial tumor infiltration of the hepatic artery, celiac axis, or the superior mesenteric artery can be regarded as a symptom of biologically aggressive tumor spread. Although in some patients this is considered as borderline resectable according to the ISGPS consensus statement, an upfront resection is rarely recommendable, even if it can technically be performed [10]. In general, in case of arterial tumor infiltration, a neoadjuvant treatment should be evaluated to achieve a better local tumor control. This treatment can be performed following different study protocols and is not standardized yet. In many protocols, gemcitabine is combined with a 50–60 Gy radiation over a 6-week period, followed by 4–6 week interval to await downsizing and development of fibrosis as a consequence of the therapy [43]. After restaging, patients should be subjected to surgical exploration as long as no signs of systemic tumor spread are visible. Using this approach, in 33–50% of all primarily irresectable patients, a radical resection is possible which achieves R0 resection rates comparable to standard resections [44–47]. To clarify arterial infiltration along the superior mesenteric artery intraoperatively, the “artery-first” approach is a useful procedure [48]. Preparation starts at the superior mesenteric artery as the initial step before further mobilization of the pancreatic head. The preparation is carried out with the incision of the peritoneal layer at the ligament of Treitz from the left side and continued by clearing the tissue along the artery down to the origin from the aorta via this access. Tumor infiltration can be ruled out or confirmed by this preparation to determine the further procedure.

In a recent review, the role of arterial resection has been critically evaluated [49]. Besides this left-sided inframesocolic “artery-first” approach, various other techniques have been published starting from arterial preparation on the right side or from a supracolic approach [50–54]. Regarding resection of the superior mesenteric artery, only five studies were available, including a total number of less than 30 patients. All authors showed that the resection is technically possible; grafting with the saphenous vein was the most commonly used method for reconstruction. However, morbidity of this approach is high and the oncological outcome is not yet convincing from the limited evidence.

Celiac axis or hepatic artery resection is performed more often. The available literature on this topic includes approximately 200 patients [55, 56]. Surgical morbidity is up to 40%; mortality in pancreatoduodenectomy with arterial resection ranges from 0 to 35%, showing the inconsistent data basis of this approach. Outcome in terms of oncological results seems to justify the approach especially in distal pancreatectomy [57] as long-term survival seems to be nearly equal to the standard approaches. However, it must be clearly stated that arterial resection does not represent a standard procedure but has to be based on an individual decision of an experienced pancreatic surgeon.  Table 2 summarizes selected studies on arterial resections.

From the technical point of view, when arterial resection is performed, resection without reconstruction has to be differentiated from resection with direct anastomosis or graft insertion to replace the resected vessel. The celiac axis might be resected down to its aortal orifice in PD as well as in DP or TP [55–57]. As long as the proper hepatic artery can be preserved, a reconstruction is possible. The left gastric and splenic artery can usually be cut without reconstruction; a consecutive splenectomy may be necessary in some patients. Restoration of the hepatic perfusion must be ensured by reconstruction of the proper or common hepatic artery. This reconstruction can be done with an interposition of any arterial vessel of the celiac axis or a venous interposition graft. The splenic artery especially is a suitable vessel for this reconstruction, either with a transposition if the base of celiac axis can be preserved or with an interposition which requires an additional anastomosis between graft and aorta [58].

However, the arterial perfusion of the liver should be controlled by regular duplex examinations and restored aggressively in case of a vessel occlusion. Arterial hepatic perfusion failure may otherwise cause acute problems postoperatively in terms of liver ischemia, necrosis, and infection and is a risk factor for bile-duct associated complications in the long-term follow-up [59, 60]. Using this approach, arterial resection can be carried out safely in experienced hands but has to be regarded as a highly individual decision in suitable patients.

## 5. Combined Vascular Resections

**Figure 2:**
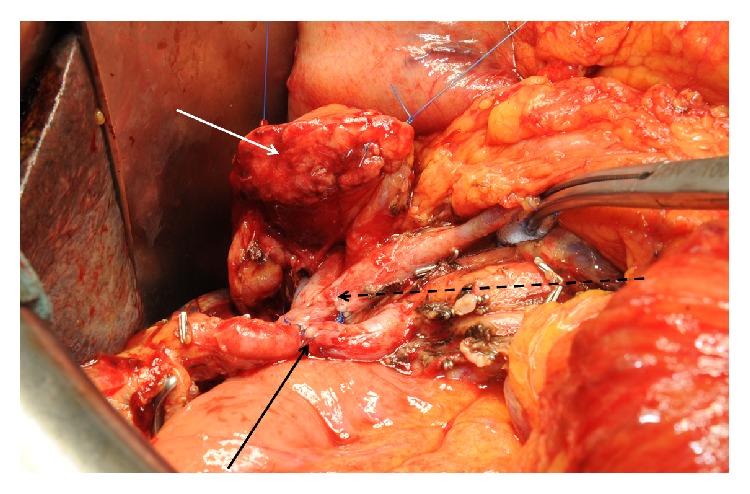
Intraoperative view in combined arterial and venous resection. Arterial anastomosis after resection of a replaced right liver artery (black arrow) infiltrated by the resected PDAC. Portal vein anastomosis (broken black arrow) and pancreatic remnant (white arrow) before completing the pancreatojejunostomy.

A combination of both venous and arterial vessel resections is technically possible in selected patients. Comparable to arterial resections alone, this approach is not recommended as a standard procedure but has to be based on individual decisions. As it has been performed in only small patient series to date, there is only very limited literature published on this topic; no conclusive evidence with regard to perioperative morbidity and oncological outcome is available. Combined vascular resections may be an individual option for patients that are considered suitable in terms of age and comorbidities (Figure 2). As this approach may be associated with a considerable surgical morbidity and even mortality as well as impaired postoperative quality of life compared to standard pancreatic operations, it must not only be evaluated with focus on technical feasibility but also on quality-adjusted lifetime that may be gained by its performance. As these extensive resections may be performed during total or even multivisceral pancreatectomies, side-effects of these approaches may be aggravated. Intestinal discomfort, diarrhoea, and food intolerance especially have to be taken into account as long-term consequences that may impair patients' performance status and especially suitability for adjuvant therapies. However, when a radical resection can be performed, present data support this concept in young and otherwise healthy patients as, once the postoperative course is completed, the prognosis from the oncological point of view seems to be superior to any palliative treatment option. Future studies in growing patient collectives will add evidence to these topics.

## 6. Conclusions

In conclusion, vascular resections in PDAC surgery are extended approaches and can be performed in many situations. The recent ISGPS consensus definitions of borderline resectability and extended resections will help to standardize these procedures in the scientific reporting in the future and make studies on this topic more comparable. Venous resections should routinely be performed when there are no other contraindications for surgery in the respective patients and can also be combined with multivisceral approaches with good surgical and oncological outcome which has also been clearly stated in national and international guidelines in the meantime. Arterial resections might be justified in selected cases after careful evaluation of the risk-benefit ratio for the individual patient. In the majority of patients, however, an evident arterial infiltration should primarily be treated by neoadjuvant therapy and reevaluated for a possibility of surgery afterwards. All surgical approaches must be part of interdisciplinary multimodal concepts as radical resection alone cannot achieve optimal patient outcome and always needs to be followed by adjuvant treatment.

## Figures and Tables

**Table 1:** Series with >50 patients comparing resection for pancreatic cancer with and without mesentericoportal vein resections 1995–2015.

Author, year	Patients PVR/no PVR	OP time (min)	R0 rate (%)	Morbidity (%)	Mortality (%)	Survival (months)
Harrison et al., 1996 [13]	58/274	444/348	74.1/76.3	nm	5.0/3.0	13.0/17.0 (median)
Hartel et al., 2002 [14]	68/203		61.8/73.4	27.0/22.0	4.0/3.0	
Riediger et al., 2006 [15]	53/169	500/440	69.0/79.0	23.0/35.0	3.8/4.1	15.0% (5 years)
Ouaissi et al., 2010 [16]	59/82	480/420	57.6/86.6	52.5/54.9	1.7/1.2	17.5/18.7 (median)
Banz et al., 2012 [17]	51/275	nm	49.0/63.3	27.5/28.4	13.7/5.1	14.5/14.8 (median)
Murakami et al., 2013 [18]	61/64	nm	50.8/71.9	36.1/21.9	0.0/0.0	14.7/26.7 (median)
Ravikumar et al., 2014 [19]	230/840	300/250	37.1/48.4	34.3/30.8	4.6/4.2	18.2/18.0 (median)
Kulemann et al., 2015 [20]	131/208	463/427	64.6/76.2	55.7/50.0	3.3/5.1	21.6/19.7 (median)

nm: not mentioned.

**Table 2:** Series of arterial resections for pancreatic cancer.

Author, year	Patients	OP time (min)	R0 rate (%)	Morbidity (%)	Mortality (%)	Survival (months)
Stitzenberg et al., 2008 [21]	12	660	50.0	100.0	17.0	17
Wang et al., 2008 [22]	19	nm	nm	36.8	0.0	16.0% (1 year)
Sugiura et al., 2009 [23]	26	nm	nm	nm	nm	10% (5 years)
Hartwig et al., 2009 [24]	14	450	57.4	37.6	6.9	nm
Ouaissi et al., 2010 [16]	8	570	50.0	75.0	12.5	11.0 (median)
Yamamoto et al., 2012 [25]	13	620	31.0	92.0	0.0	20.8 (median)
Yoshidome et al., 2014 [26]	7	522	nm	29.0	0.0	12.7 (median)

nm: not mentioned.
